# Trace Metal Accumulation in Rats Exposed to Mine Waters: A Case Study, Bor Area (Serbia)

**DOI:** 10.3390/toxics11120960

**Published:** 2023-11-25

**Authors:** Ion Valeriu Caraba, Marioara Nicoleta Caraba, Delia Hutanu, Adrian Sinitean, Gabi Dumitrescu, Roxana Popescu

**Affiliations:** 1 Faculty of Bioengineering of Animal Resources, University of Life Sciences “King Mihai I” from Timisoara, Calea Aradului, 119, 300645 Timisoara, Romania; caraba_i@animalsci-tm.ro (I.V.C.); gdumitrescu@animalsci-tm.ro (G.D.); 2 Department Biology-Chemistry, Faculty of Chemistry, Biology, Geography, West University of Timisoara, Pestalozzi 16, 300315 Timisoara, Romania; delia.hutanu@e-uvt.ro (D.H.); adrian.sinitean@e-uvt.ro (A.S.); 3 ANAPATMOL Research Center, Faculty of Medicine, “Victor Babes” University of Medicine and Pharmacy Timisoara, 300041 Timisoara, Romania; popescu.roxana@umft.ro

**Keywords:** mining wastewaters, Bor mining area, trace metals, water contamination, environmental risk

## Abstract

Zinc (Zn), copper (Cu), iron (Fe), manganese (Mn), cadmium (Cd), and lead (Pb) levels were measured in the Bor City water supply system (control) and two watercourses exposed to mining wastewaters, i.e., the Lutarica River (one site) and the Kriveljska River (two sites). The same parameters were determined in the brain, heart, lungs, stomach, liver, spleen, kidneys, and testes of male Wistar rats given water from these sources for 2 months. Water Cu, Fe, Cd, and Pb were outside the safe range, excepting the reference site. Significant impacts on intra-organ metal homeostasis were detected, especially in the brain, stomach, kidneys, and testes. The dynamics and magnitude of these changes (versus controls) depended on the target organ, analyzed metal, and water origin. The greatest number of significant intra-organ associations between essential and non-essential metals were found for Cd-Zn, Cd-Cu, and Cd-Mn. A regression analysis suggested the kidneys as the most relevant organ for monitoring water manganese, and the stomach and brain for lead. These results highlight the environmental risks associated with mining wastewaters from the Bor area and could help scientists in mapping the spatial distribution and severity of trace metal contamination in water sources.

## 1. Introduction

Mining-derived waters, including mine effluents and seepage from waste rock impoundments and tailings, can easily infiltrate into surface waters and ground waters, altering the quality of water in ponds, lakes, rivers, and reservoirs [1,2,3]. The presence of elevated levels of trace metals (TMs) in drinking water sources is of worldwide concern since these elements are stable, non-biodegradable, biomagnifiable, and persistent contaminants, which can perturb physiological functions in living organisms [4,5]. In spite of extensive knowledge of the impact of TM-contaminated mining waters on terrestrial and aquatic ecosystems in Europe, there is still a lot of work to be done on this topic, especially in areas of intense mining activity from the former communist countries of Eastern and Central Europe [6]. A high-profile example is the Bor mining area in Serbia—one of the most polluted countries in Europe according to the Global Alliance for Health and Pollution [7]. Of particular importance is the long-term TM contamination (pollution) of rivers and underground waters in this region since all major local watercourses flow into the Danube River [7,8]. Several studies have analyzed the potential health hazard of these waters, but most of them have focused on their physico-chemical properties and rarely investigated their effects on toxicologically relevant mammalian models [9,10,11,12,13,14]. In fact, only one study has addressed the latter topic and used rats as a study system [4]. Such models enable scientists to realistically estimate the potential health risks associated with ingestion of TM-contaminated water since rodents are synanthropic species that share the same basic physiology with humans and other land mammals; hence, they are exposed to many of the same factors [15,16].

Exposure of land mammals to TMs occurs primarily via food consumption and water uptake, with respiration of atmospheric air serving as a secondary path [17,18,19]. Zinc (Zn), copper (Cu), iron (Fe), manganese (Mn), cadmium (Cd), and lead (Pb) are major environmental contaminants due to their broad range of industrial usage, long history of exploitation, and potential toxicological adverse effects [18]. Although Zn, Cu, Fe, and Mn function as essential TMs when given at physiological concentrations [17], both excess and deficiency are damaging to organ systems [17]. For example, zinc depletion can impair immune function, with overload reducing high-density lipoprotein (HDL) and copper levels [8,18]. Copper deficiency is associated with anemia, cardiac arrhythmia, and thyroid problems, while excess can lead to heart failure and kidney damage [1,17]. Both iron deficiency and iron excess can promote oxidative stress, impairing mitochondrial metabolism and respiratory activity [18]. Manganese excess inhibits mitochondrial function, lowers glutathione levels, and disrupts calcium homeostasis, whereas deficiency can lead to impaired reproductive function, skeletal abnormalities, and altered glico-lipidic metabolism [3,17]. In contrast, Cd and Pb are non-essential TMs, with no known vital or beneficial effect on mammals [5,17,18,19].

Environmental studies provide comprehensive evidence for the existence of area- and industry-specific TM contamination/pollution patterns [6,17,18]. In the case of the Bor mining area, this involves elevated levels of Zn, Cu, Fe, Mn, Cd, and Pb in soil and water [7,8,9,10,11,12,13,14]. All these metals can accumulate to substantial concentrations in humans and different animal models [19,20,21,22]. The identification and quantification of types and amounts of metal ions in biological samples (i.e., metallomic fingerprinting) can help identify over-physiological-exposure to essential TMs or exposure to toxic levels of non-essential TMs [18]. Such results enable scientists to determine species that may be at risk for TM toxicity and guide interventions to reduce exposure [8]. Another important aspect related to TM exposure is perturbation of intra-organ mineral homeostasis [18,20,21]. Since this process involves a delicate equilibrium between essential TMs and non-essential TMs, any imbalance can result in physiological changes and adverse health effects [17,18]. Importantly, metal–metal interactions can potentiate or mitigate the risks associated with TM exposure [18]. Knowledge of metal–metal interactions is hence important for a critical evaluation of TM levels in organs and tissues with respect to the body burden with environmental TMs [17,18]. Moreover, most TMs do not accumulate to a similar degree in all organs but are usually retained in one or two organs [17,21]. The liver and kidneys generally serve as the primary storage sites for most TMs [18], but this pattern can be affected by several factors, including exposure parameters (e.g., route, dose, duration, origin), animal model used, geogenic TM sources, metal–metal interactions across different trophic levels, and TM bioavailability [17,20,22]. It is therefore conceivable that target organs for different TMs can differ depending on location.

In this context, we hypothesized that consumption of TM-contaminated waters from the Bor area will affect the metallomic profile and intra-organ TM homeostasis across different organs in native fauna from the Bor mining area. Atomic absorption spectrometry (FAAS) was used to determine Zn, Cu, Fe, Mn, Cd, and Pb concentrations in water sources at four sites impacted to different extents by anthropogenic activities, and in selected organs of male Wistar rats. The brain, heart, lungs, stomach, liver, spleen, kidneys, and testes are known to be sensitive to the changes in levels of environmental TMs [4,17,18], and hence were the organs chosen to be investigated in this study. Intra-organ metal–metal interactions were studied via a correlational analysis. Linear regressions were used next to determine the main target organs for different TMs. The results of the present work should help inform policies and practices aimed at minimizing the adverse effects of water TM contamination on ecosystems and public health in the Bor area.

## 2. Materials and Methods

### 2.1. Study Area

**Figure 1:**
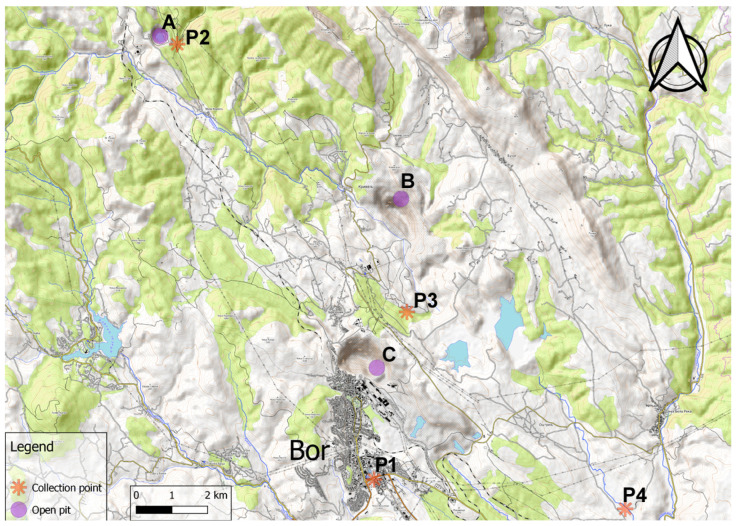
Map of the collection sites in the Bor area (Serbia). P1, Bor City water supply system; P2, Lutarica River; P3, Kriveljska River; P4, Kriveljska River; A, Open Pit Cerovo; B, Open Pit Krivelj; and C, Open Pit Bor.

Water samples (triplicates) were collected in April 2020 from four sites; their location is given in Figure 1. The reference samples were collected from the P1 site (Figure 1), i.e., the Bor City water supply system (latitude = 44°3′24.99″ N, longitude = 22°6′20.28″ E). At the P2 site (latitude = 44°9′57.99″ N, longitude = 22°2′12.24″ E), samples were taken from the Lutarica River, upstream of its confluence with the Deljboca River (Figure 1). The Open Pit Cerovo lies on the right side of the Lutarica River (Figure 1). The other samples were obtained from the Kriveljska River; more precisely, at the P3 site (latitude = 44°5′56.38″ N, longitude = 22°7′1.59″ E) and the P4 site (latitude = 44°2′58.52″ N, longitude = 23°11′36.61″ E). The former site is located downstream of the Open Pit Krivelj (Figure 1), whereas the latter site lies downstream of the Open Pit Krivelj and Open Pit Bor, farther away than the site P3 (Figure 1). These rivers are regularly exposed to mining wastewaters discharged from the surrounding open pits, being among the most polluted watercourses in Europe [4,8,12,14,23].

### 2.2. Study Animals

The present study complied with the conditions stipulated in the ethical approval obtained from the Human Research and Ethics Committee of the West University of Timisoara (Ethic Approval no. 347/28, April 2020). These conditions are in agreement with the Directive 2010/63/EU of the European Parliament and of the Council of 22 September 2010 on protection of animals used for scientific purposes [24]. Test animals (40 adult male Wistar rats) were obtained from the Faculty of Bioengineering of Animal Resources, “King Michael I” University of Life Sciences (Timişoara, Romania). The rodents were 24 weeks of age, with a mean weight of 425 ± 22 g (g) and a mean length of 242 ± 28 mm (mm). During the 2-month experimental period, the rodents were kept in individual cages in the Laboratory of Anatomy, Histology, and Embryology under standard conditions of temperature (25 °C), humidity (45–55%), and photoperiod (12 h:12 h light–dark cycle).

After being allocated to four groups of 10 specimens each, the male Wistar rats were fed a normal diet (standard rodent chow) and given drinking water ad libitum from the aforementioned sites (renewed daily). Ad libitum water drinking implies that the rodents consumed water whenever, and in whatever volume, desired. This strategy is routinely used in exposure experiments for chemicals in water [25]. At the end of the experiment, the rats were water- and food-fasted overnight prior to sacrifice. This aimed to minimize the interference from recent dietary intake on TM levels in different organs. Per each treatment group, three randomly chosen specimens were weighed on an analytical balance and then anesthetized using 100 parts per million (ppm) ketamine and 20 ppm xylazine. After performing an abdominal incision along the linea alba, each animal was sacrificed using exsanguination and triplicate samples were collected from the brain, heart, lungs, stomach, liver, spleen, kidneys, and testes. All samples were stored at −20 °C prior to a chemical analysis.

### 2.3. Chemical Analyses

The levels of Zn, Cu, Fe, Mn, Cd, and Pb in water samples were determined by using a flame atomic absorption spectrophotometer with a high-resolution continuum source (Model ContrAA 300, Analytik Jena, Jena, Germany), fitted with specific conditions for each metal. The experimental protocol is similar to those used in our previous investigations [3,4]. More precisely, the sediment recovered after water evaporation was dissolved in 20 milliliters (mL) of a 0.5 N HNO_3_ solution and filtered through ash-free filter paper prior to the chemical analysis. For each sample, the volume was brought to 50 mL with 30 mL of the 0.5 N HNO_3_ solution. The trace metal grade, the concentrate nitric acid (HNO_3_, 65%), used to prepare digestion solutions was obtained from Merck Group (Sigma-Aldrich Chemie GmbH, Buchs, Switzerland).

Animal tissue samples were weighed with the analytical balance (Kern model ALJ 220-4NM, Denver Instrument Gmbh, Göttingen, Germany) (5 g/each organ) and then dried at 105 °C for 48 h (thermal oven from Memmert GmbH, Schwabach Germany). These samples were digested in a calcination furnace (Nabertherm Controller B170, Lilienthal, Germany), in which the temperature progressively increased until 550 °C. After 4 h, the white ash obtained was dissolved in 20 mL of the 0.5 N HNO_3_ solution and filtered through a paper filter. Mix standard solutions of Fe, Mn, Zn, Cu, Ni, Cd, and Pb—ICP Multi-element Standard solution IV CertiPUR—were purchased from Merck Germany. Stock solutions (1000 ± 5 ppm) for each analyzed TM were obtained from May & Baker Group PLC (Lagos, Nigeria) and prepared in three different concentrations for constructing the corresponding calibration curves.

All glassware was treated with a Pierce solution at 20% (*v*/*v*), rinsed with cold tap water, treated with 20% (*v*/*v*) nitric acid, and then rinsed again with double distilled water. All blanks and duplicate samples were analyzed during the procedure. NCS Certified Reference Material—DC 85104a and 85105a (China National Analysis Center for Iron & Steel)—was used for quality assurance. Percentage recoveries for the TM analysis varied between 85 and 105%. Percent recovery averages were Zn (102%), Cd (105%), Cu (105%), Fe (92%), Mn (95%), and Pb (94%). Variation coefficients were below 10%. Detection limits (ppm) were assessed via the calibration curve method: Zn (0.43), Cd (0.01), Cu (0.13), Fe (0.15), Mn (0.19), and Pb (0.05). TM levels in water samples were expressed as parts per million TM dry weight (ppm). All measurements were performed by the same researcher in the same conditions for all sampling sites and seasons.

### 2.4. Statistical Analyses

Inter-group differences in post-exposure body weight were analyzed performing a one-way ANOVA. A similar approach was used for determining differences in organ TM concentrations (as log_10_-transformed data sets). In case of significant differences, Dunnett’s tests were employed for post hoc comparisons against controls. Pearsons’s correlations (*r*) were next applied on pooled data sets (for all treatment groups) to identify the patterns of intra-organ metal–metal interactions. The strength of these associations was described as weak, *r* = 0.31–0.50; moderate, *r* = 0.51–0.69; and strong, *r* = 0.70–1.00 [26].

A possible linear relationship between TM levels in water and organs analyzed was checked through Pearsons’s correlation coefficients (*r*), with the strength of associations being determined as described above. For significant correlations, simple linear regressions were applied with TM concentration in organs as the dependent variables and the values measured in the water as the independent variables. The corresponding coefficients of determinations (R^2^) allowed us to assess the proportion of variation in organ TM levels that is predictable from changes in water TM concentrations. An R^2^ > 0.8 was considered as an indicator of a good linear fit; 0.5 ≤ R^2^ ≤ 0.8, a moderate fit; and R^2^ < 0.5, a weak fit [26]. All statistical analyses were run using Statistica version 8 software (StatSoft Inc., Tulsa, OK, USA). Statistical significance was defined at *p* ≤ 0.05.

## 3. Results

The measured values for body weight at the end of the experiment were similar between the P1 rats (438 ± 22 g), the P2 rats (442 ± 35 g), the P3 rats (431 ± 32 g), and the P4 rats (435 ± 27 g). No significant inter-group differences were found for body weight (ANOVA, *p* = 0.752). Moreover, no mortalities were recorded during the 2-month exposure period.

### 3.1. Water TM Levels

Average TM concentrations in water sources are shown in Table 1. Copper, iron, manganese, and lead levels varied widely between the sites analyzed (Table 1). However, the measured values increased in the same order, P1 site < P2 site < P3 site < P4 site, and differed by at least two orders of magnitude between the P2, P3, and P4 sites and the reference site. Zinc concentrations revealed a pattern of variation similar to that seen for the aforementioned TMs (Table 1); the greatest levels were found in the P4 water although the magnitude of difference *versus* the tap water from the Bor City water supply system (P1 site) was much smaller. In contrast, cadmium in drinking water had the lowest value for the P1 site and the P3 site, followed by the P2 site and the P4 site (Table 1).

### 3.2. Effect of Drinking Water Sources on Intra-Organ TM Homeostasis

**Figure 2:**
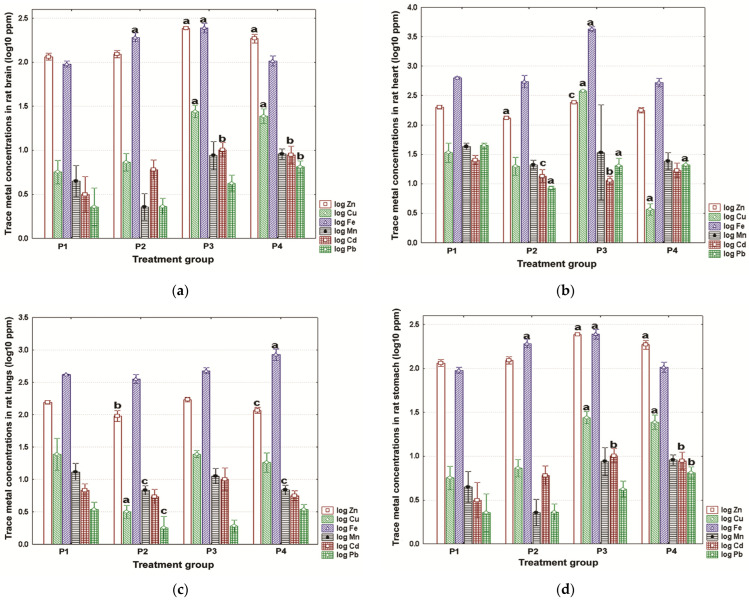
Mean content of zinc, copper, iron, manganese, cadmium, and lead in (**a**) brain; (**b**) heart; (**c**) lungs; and (**d**) stomach. Data were calculated for three technical replicates (triplicates) and three biological replicates (*n* = 3) per treatment group. The measured values are expressed as parts per million dry weight (ppm) and are shown on a log_10_ scale as mean (box) with one standard deviation (error bar). Marked boxes indicate significant differences as compared to the reference group (Dunnett’s test, ^a^—*p* < 0.001, ^b^—*p* < 0.01, ^c^—*p* < 0.05).

The brain, stomach, kidneys, and testes showed significant inter-group differences irrespective of TMs analyzed (ANOVAs, *p* ≤ 0.016). This trend was seen for the other organs (tissues) analyzed (ANOVAs, *p* ≤ 0.03), except for Zn in the spleen and muscles; Fe in the spleen; Mn in the heart and lungs; and Cd in the lungs, heart, and liver (ANOVAs, *p* ≥ 0.076). The levels of TMs in the rat brain, heart, lungs, and stomach are shown in Figure 2a, Figure 2b, Figure 2c, and Figure 2d, respectively.

Altered homeostasis of brain TMs was particularly evident for zinc and iron, with significant differences *versus* controls being detected for all experimental groups (Figure 2a). The most obvious changes were seen for the P4 rodents, that is, significantly elevated Zn, Mn, and Pb levels, but significantly decreased Fe retention (Figure 2a). Lead disclosed the most homogeneous pattern of changes in the heart of male Wistar rats, showing significantly decreased concentrations irrespective of treatment group (Figure 2b). Specimens given the P3 water exhibited the most evident dysregulation of cardiac homeostasis, revealing significantly increased Zn, Cu, and Fe and significantly decreased Pb and Cd compared to the reference group (Figure 2b).

The most noticeable pulmonary effects were Zn, Fe, Cu, and Mn imbalance (Figure 2c). Significantly reduced levels were detected for zinc, copper, and manganese in the P2 specimens (Figure 2c). Similar trends were observed for Zn and Mn in the P4 rats, but lung iron was significantly elevated (Figure 2c). Stomach TMs tended to be elevated in the P2, P3, and P4 rodents (Figure 2d). Zn, Cu, and Cd in the P3 rats and P4 rats revealed the most perturbed homeostasis, being significantly enriched compared to controls (Figure 2d). A significant Pb excess in the stomach of P4 rodents was also identified (Figure 2d).

**Figure 3:**
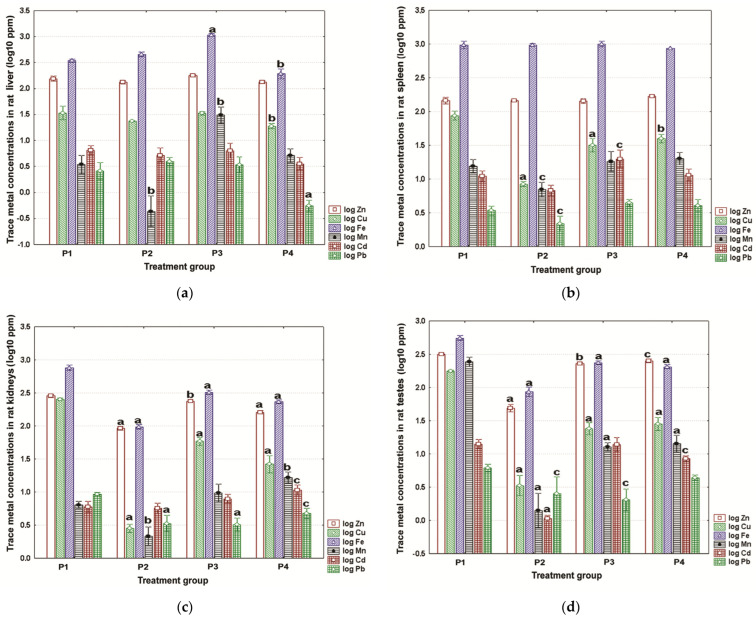
Mean content of zinc, copper, iron, manganese, cadmium, and lead in (**a**) liver; (**b**) spleen; (**c**) kidneys; and (**d**) testes. Data were calculated for three technical replicates (triplicates) and three biological replicates (*n* = 3) per treatment group. The measured values are expressed as parts per million dry weight (ppm) and are shown on a log_10_ scale as mean (box) with one standard deviation (error bar). Marked boxes indicate significant differences as compared to the reference group (Dunnett’s test, ^a^—*p* < 0.001, ^b^—*p* < 0.01, ^c^—*p* < 0.05).

The concentrations of TMs in the liver, spleen, kidneys, and testes are depicted in Figure 3a, Figure 3b, Figure 3c, and Figure 3d, respectively. The liver was one of the organs (tissues) least affected by drinking water origin (Figure 3a). The largest effects were significant Fe and Mn increases in the P3 rats, and significant Cu and Fe decreases in the P4 rats (Figure 3a). There was also a significant reduction in Pb concentrations in the latter experimental group (Figure 3a). In the case of the spleen, copper was significantly lower for all groups analyzed (Figure 3b). Male rats given the P2 water revealed the most noticeable differences in TM content versus controls, more precisely, a significant depletion for Cu, Mn, and Pb (Figure 3b).

There was a strong effect of water origin on TM retention in the kidneys (Figure 3c) and testes (Figure 3d). For the former organ, most TMs tended to show significant differences compared to controls (Figure 3c). The most consistent patterns of changes were seen for zinc, copper, iron, and lead, with the measured values being significantly reduced in all experimental groups (Figure 3c). The magnitude of changes was the highest in the P2 rats (Figure 3c). The same tendency towards decreased TM levels was also noticed in the testes (Figure 3d). The pattern of changes was comparable to that observed for the kidneys; that is, Zn, Cu, Fe, and Mn showed the most evident changes, with the P2 rodents being the most affected group (Figure 3d).

### 3.3. Intra-Organ TM Correlations

Table 2 shows the correlations between the TM levels in different organs. Copper concentrations in the brain were strongly negatively correlated with the measured values for zinc and iron (Table 2). The latter TM displayed a similar relationship with brain manganese content (Table 2). Lead, in contrast, revealed significant positive associations with both Mn and Cd. The strength of such relationships was generally lower in the heart of male Wistar rats. Thus, Zn showed moderate relationships with Cu, Mn, and Cd contents (Table 2). In contrast, cardiac iron was strongly correlated with cardiac copper (Table 2). Weaker associations were found in the lungs of male rodents. Zinc correlated moderately to strongly with copper, manganese, and cadmium (Table 2). Mn concentrations showed a moderate positive relationship with lung Cu (Table 2).

The stomach showed a higher number of significant correlations among TM levels (Table 2), with most associations being strongly positive (Table 2). Such relationships were observed between zinc and copper, manganese, cadmium, and lead; copper and manganese, cadmium, and lead; and manganese and lead (Table 2). Liver zinc correlated positively with copper, iron, and manganese concentrations (Table 2). Similar relationships were seen for Cu and both Fe and Cd, and between Fe and Pb (Table 2). Zinc and iron content of the spleen was strongly negatively correlated (Table 2). Strong but positive associations were identified for Pb-Mn and Pb-Cd interactions (Table 2). There were also moderately positive correlations between Mn levels and both Cu and Cd concentrations in the rodent spleen (Table 2).

Renal Zn was strongly positively correlated with the measured values for Cu and Fe (Table 2). Similar relationships were identified between copper and both iron and lead (Table 2), as well as between manganese and cadmium (Table 2). In contrast, there was a negative association between iron and lead and a weaker but direct association between Cu and Pb (Table 2). Rodent testes showed the highest number of significant inter-organ metal–metal relationships and the majority of them were strong direct correlations (Table 2). This was the case of Zn-Cu, Zn-Fe, Zn-Mn, Zn-Cd, Cu-Fe, Cu-Mn, Mn-Cd, Fe-Mn, Fe-Cd, and Mn-Cd associations (Table 2).

### 3.4. Water-to-Organ Linear Regressions

The results of Pearson’s correlation analysis for TM levels in water and different organs are given in Table 3. The highest number of significant correlations was found for the brain, stomach, and kidneys of male Wistar rats (Table 3). In contrast, no significant associations were identified for the heart and lungs (Table 3). Strong positive correlations existed between Zn, Mn, Cd, and Pb levels in water and the rat brain (Table 3). Stomach zinc, copper, manganese, and lead revealed similar relationships with the values measured in water (Table 3). The liver displayed moderately and highly negative associations for cadmium and lead (Table 3). The only significant association in the case of the spleen was found for Mn (Table 3). The kidneys exhibited moderate to strong negative associations for Cu and Fe, but inverse relationships for Mn and Cd (Table 3). Testicular iron correlated negatively with water Fe content (Table 3).

The values of coefficients of determination derived from the linear regression analysis of TM levels in water and different organs are given in Table 3. For zinc, the best fit was identified for water-to-brain regression (Table 3). The increase in water copper was best predicted by an increase in stomach Cu content, the magnitude of change being close to that described for Zn (Table 3). For Fe, Mn, and Cd, the best fits were detected for water-to-kidney regressions, with the highest R^2^ value being observed for manganese (Table 3). The stomach and kidneys were the most sensitive target organs with respect to their overall response to changes in water TM content (Table 3). Moreover, the increases in lead concentrations in the brain and stomach were good predictors for an increase in water lead content (Table 3).

## 4. Discussion

This is the first study to establish a link between TM-contaminated water sources from the Bor mining area and metal dyshomeostasis in mammals. By investigating TM retention and distribution across eight tissues, this paper also substantially expands previous knowledge on the environmental hazard of these water sources, which until now was limited to quantitative data derived from only one study [4]. Moreover, the results of the current investigation provide pertinent information on the primary target organs for TMs specific to the contamination/pollution pattern of the Bor mining area.

### 4.1. Water TM Levels

Essential TMs were outside the recommended safe range in drinking water samples collected from the Lutarica River, and Kriveljska River, that is, 5, 1.3, and 0.05 ppm for zinc, copper, and manganese, respectively [27,28,29]. Water iron was above the maximum permissible limits, i.e., 0.3 ppm [28], irrespective of site. However, levels of up to 10 ppm—like those measured in tap water from the Bor City water supply system—are considered safe for human consumption since this standard refers to water appearance, staining, and taste, not to the potential adverse health effects [28,29]. With respect to non-essential TMs, water cadmium was relatively low, lying within the accepted range, i.e., 0.1–1 ppm [27]. Importantly, the measured values in tap water from the Bor City water supply system were below the value protective for human health when using non-carcinogenic endpoints of toxicity to assess the risk over lifetime of exposure, i.e., 0.02 ppm [28]. Since the lead content was also close to the current maximal accepted limit of 15 parts per billion (ppb) [28,29], this tap water seems safe for human/animal consumption in terms of Cd/Pb content. The other water sources, in contrast, showed Pb concentrations up to 500-fold higher than the aforementioned threshold. These elevated levels of Cu, Fe, and Pb are consistent with previous results on TM contamination/pollution of water sources from the Bor area [11,12,13,14,30,31]. These findings raise serious concerns about the environmental hazard posed by non-ferrous metal mining and processing operations in this area.

### 4.2. Effect of Drinking Water Sources on Intra-Organ TM Homeostasis

In vivo rodent studies are often used to investigate the transfer (bioavailability) of TMs (mixtures of TMs) from contaminated (polluted) water sources in mammals and determine their distribution across different organs [16,17]. One approach is to use the bioaccumulation factor (BAF)—calculated as the ratio between TM concentrations in organs and water [17,18]. However, direct measurement of TM levels in mammalian organs offers several advantages over such indices. First, this approach allows for species- and organ- specific assessment of TM accumulation and provides accurate information on the specific tissues/organs where TM accumulation is preferentially occurring [17]. Second, it can detect even low levels of TM accumulation, which may not necessarily result in mortality but could lead to sublethal effects and chronic health problems [17]. Third, direct measurement avoids certain assumptions underlying the use of BAF and similar indices, such as steady-state conditions, linear accumulation, no metabolism or detoxification, homogeneous environment, or single TM consideration [18]. As a result, this approach provides a more informative and comprehensive approach in exploratory studies [17], such as was the case with our investigation.

Despite an overall trend towards significant differences in organ TM levels *versus* controls, spleen Zn, Fe, and Mn, as well as cardiac Fe and Mn, were not affected by the drinking water origin. These effects may reflect the important roles of these essential TMs for the proper functioning of the spleen and heart. For example, Zn is a potent antioxidant, with deficiency being associated with spleen enlargement [32]. Moreover, the spleen stores Fe as ferritin/bilirubin before returning it to the bone marrow for producing hemoglobin [33]. For non-essential TMs, no changes were seen for Pb and Cd levels in the lungs and heart, as well as for liver cadmium. The latter observation is intriguing since the liver is an important target for cadmium accumulation and toxicity [34,35]. Low cadmium levels in water sources studied here may account for these findings.

Iron or zinc dyshomeostasis in the brain, as observed in all treatment groups, can yield serious neurological consequences. Low brain Fe causes decreased neuronal activity and increased anxiety-like behaviors [36]. On the other hand, iron excess induces neuronal damage, being linked to the development of neurodegenerative conditions [37]. In the case of Zn, deficiency, rather than excess, is potentially damaging for the brain. For example, zinc depletion impairs learning and memory performance in rats [38,39]. Manganese excess or lead excess, as detected in the P4 rats, can perturb the brain neurotransmitter systems and respectively reduce brain weight in rodents [40,41].

The most consistent cardiac outcome was the significant depletion of lead for all treatment groups. Because this TM can affect cardiac and hematological parameters in mammals [42,43], the P3 specimens may display a lower cardiovascular Pb-related risk. However, this advantage may be counterbalanced by significant zinc, copper, and iron elevations. Thus, excess Zn suppresses rat myocyte beat frequency in vitro [44], being in humans related to several types of heart disease [45]. Copper overload induces cardiac hypertrophy, ischemic disease, and fibrosis in both rodents and humans [46]. In addition, iron excess in the male rat heart yields oxidative damage and cardiomyopathy [47].

TM-contaminated drinking waters exerted a moderate impact on pulmonary metal homeostasis. This may reflect the fact that TM exposure via water serves as a minor exposure path [48]. Based on the present findings, the P2 rats and P4 rats appeared to be the most affected specimens, showing a simultaneous decrease in pulmonary Zn and Mn. Zinc is essential for the respiratory epithelium due to its roles in immune function, antioxidant defense, mucociliary clearance, and epithelial integrity. Its deficiency in alveolar macrophages and lung epitheliums decreases lung barrier functionality [49]. Manganese imbalance may also affect lung functions since this TM helps form superoxide dismutase (SOD), which is essential for protecting the lungs against oxidative stress [50].

Elevated Cu, Cd, and Pb concentrations, as found in the stomach of the P3 and P4 rats, are known risk factors for gastric diseases, including gastric cancer [51,52]. TM levels in blood and its derivatives are reliable indicators of gastric TM content [19]. The aforementioned findings can hence be pertinently interpreted based on the toxicological data linking them to stomach cancer. For example, serum copper levels correlated directly with the incidence of this cancer type [53]. Blood Cd and Pb were significantly higher in individuals with gastrointestinal cancers compared to healthy controls [54]. Moreover, the incidence of stomach cancer deaths in the UK tended to increase with water Pb contamination [55]. Nonetheless, high zinc levels—as observed in the P3 and P4 rats—may attenuate this risk since zinc is protective against stomach cancer [56].

The liver was less affected by the TM water content, but simultaneous disbalance of hepatic Fe and Mn was evident for the abovementioned groups. Both hepatic iron excess, as seen in the P3 specimens, and depletion, as detected in the P4 rats, can cause mitochondrial dysfunction and impair the glico-lipidic control [57,58]. Glico-lipidic imbalance can also occur in the case of copper depletion, as observed in the P4 rats, finally leading to non-alcoholic fatty liver disease [59,60]. Moreover, manganese accumulation not only causes liver dysfunction, but the excess not excreted into the bile is transported to the central nervous system, causing neurotoxicity [61].

The most noticeable outcome in the rat spleen was significant reduction in Cu concentrations in TM-exposed specimens. Splenic hypocupremia can impair neutrophil function and alter splenocyte T/B cell responsiveness [62,63]. This decreased immune response is of particular concern for the P2 rats; these rodents revealed low manganese levels—a key enhancer of splenic inflammatory response in the fight against pathogens [64].

An obvious trend of decreasing renal zinc, copper, iron, and lead was identified for all treatment groups. Such changes are associated with reduced kidney function and chronic renal conditions [64]. Thus, Zn deficiency in rats activates renal interstitial fibroblasts and reduces the expression of fibrosis-associated factors via the TGF-β/Smad signaling pathway [65]. Low copper concentrations decrease ceruloplasmin activity, being associated with lesions in the rat renal cortex, medulla, and papilla [66]. Reduced kidney iron causes glomerular sclerosis, urinary protein excretion, and progressive tubulointerstitial damage in iron-deficient rats [67]. Moreover, low renal manganese exerts adverse kidney effects via reduced Mn-SOD activity [68].

Concomitant reduction in gonadal Zn, Cu, Fe, or Mn in the P2, P3, and P4 rats may be harmful for species survival and persistence. Zinc deficiency results in Leydig cell degeneration and loss of germ/somatic cells [69]. Testicular copper depletion reduces fertility in male rats, while also impairing Fe and Zn homeostasis [70]. In addition, low iron can alter spermatogenesis and testicular morphology [71]. Furthermore, manganese depletion can lead to complete sterility due to the lack of spermatozoa production [72].

Based on the aforementioned findings, water samples from the P2 site appear to exert the strongest impact on TM homeostasis in the lungs, spleen, kidneys, and testes; those from the P3 site on TM homeostasis in the heart, stomach, and liver; and those from the P4 site on TM homeostasis in the brain, lungs, stomach, and liver. The effects of drinking water from different locations on TM homeostasis in various rat organs are hence complex and multifaceted [17,48,73]. It is also worth noting that TM levels in certain organs were more sensitive to water TM changes compared to body weight and rat survival. Hence, these organs might serve as reliable indicators of TM exposure, as their responses are more pronounced and quicker to change compared to other physiological- and survival-related measures. If validated, these potential biomarkers could have important implications for TM risk assessment and monitoring in the Bor mining area.

### 4.3. Intra-Organ TM Correlations

Mineral homeostasis in living systems is potentiated by metal–metal interactions, which can be antagonistic or synergistic depending on their physicochemical properties (e.g., electronic structure, valence shell, redox potentialation) [8,17,48]. When the associations between essential and non-essential TMs reached statistical significance, most of them were moderately or strongly positive. The highest number of significant correlations was identified for cadmium; more precisely, for Cd-Zn, Cd-Cu, and Cd-Mn interactions. It is known that zinc and cadmium tend to bind to the same proteins in biological systems, mainly, metallothionein (MT) in tissues and albumin in blood [5,17,74]. Since Zn^2+^ and Cd^2+^ ions compete for cellular uptake and binding to intracellular sites [19,52], these ions can affect the uptake and action of each other, depending on their levels. Cadmium is eight-fold more potent than zinc in raising liver MT concentrations [19,23]; hence, any increase is likely to have a stronger impact on Zn than vice versa. A comprehensive review of Zn-Cd interactions in biological systems revealed that, as a general trend, tissular zinc increases with the Cd dose and the subsequent elevation of tissular Cd [74]. This tendency is concordant with the direction of Cd-Zn correlations seen here.

Cadmium uptake can also disrupt copper distribution in different tissues [17,18,60,75]. For example, Cd concentrations increased with Cu levels in the liver of male rats given Cd-spiked fodders [76]. These interactions are most probably again related to the aforementioned ability of cadmium to induce MT synthesis, thus perturbing the intra-organ copper homeostasis and storage [17,77]. With respect to the relationship Cd-Mn, there are very few joint toxicity data sources for the impact of such interactions on mineral homeostasis in different organs. However, consistent with our findings, renal manganese was found to increase following consumption of cadmium-enriched drinking water [78].

### 4.4. Water-to-Organ Linear Regressions

Animal organs are frequently used as exposure biomarkers for trace metals (TMs) in contaminated waters [3,17,18,25,79]. One key criterion for the effectiveness of such biomarkers is the presence of a direct linear dose–response relationship [79]. A strong relationship is often indicated by a high R^2^ value (R^2^ ≥ 0.7) and a strong positive correlation *(r* ≥ 0.7) [26]. Under the current experimental conditions, the brain, stomach, and kidneys showed the most significant responses to variations in water TM content. However, only the kidneys exhibited a strong dose–response relationship for manganese, while both the stomach and brain exhibited such responses for water lead.

The brain showed a dose-dependent trend of increasing zinc and manganese, serving as the primary target organ for the former TM. It may therefore be more susceptible to zinc toxicity [80], generally manifested via lethargy and neuronal loss [81]. In fact, zinc overaccumulation induces dopamine, norepinephrine, and epinephrine elevation in the brain of a male albino rat, *Rattus norvegicus*, resulting in mild tremors, reduced locomotor activity, and restlessness [82]. Moreover, zinc overload in a human brain is associated with onset and progression of Alzheimer’s disease [17,83].

The rat stomach showed the highest affinity for copper, while serving as an important storage site for zinc and manganese. Copper, particularly in its primary ionic form (Cu^2+^), can form soluble copper salts that are dissolved and easily absorbed in the acidic environment of the stomach [48]. Functioning as the first organ in the gastrointestinal tract that ingested copper encounters, this organ is likely to accumulate copper before it can be transported further along the digestive system [84]. This could help explain the preferential retention of copper in the stomach of male Wistar rats. However, it is worth mentioning that copper eventually passes from the stomach to the liver, wherein it is further regulated and stored in higher quantities [84]. Since the liver was not a relevant accumulator for any TMs analyzed here, it is plausible that exposure duration was too short to allow a significant increase in hepatic copper (and other essential TMs analyzed).

Although no organ served as a major sink for iron, the testes and kidneys showed a significant dose-dependent decrease in Fe concentrations. When there is an excess of iron in the diet or water, the body may prevent iron overload by decreasing the absorption of dietary Fe from the gastrointestinal tract [18], thus reducing the amount of iron available for distribution to various tissues, including the kidneys and testes [85]. In response to high Fe concentrations, the body may also increase the synthesis of iron-binding proteins like ferritin, sequestering excess iron and preventing its accumulation in tissues [86]. In addition, excess iron can compete with other metals, such as manganese, for absorption [18,48]. This competition can affect the uptake of iron into the bloodstream and, consequently, its distribution to different organs. Indeed, a significant increase in renal manganese coupled with a significant decrease in renal iron support this assumption.

The kidney served as the main target organ for manganese—an essential TM with potent nephrotoxic effects [17,20,87]. This retention pattern is most probably related to the mechanisms underlying Mn accumulation and elimination in mammals. It is thus known that excess manganese is primarily eliminated from the body through bile excretion and gastrointestinal elimination, while the kidneys function only as a minor elimination route [87]. Based on the strength and magnitude of the relationship between Mn levels in rat kidneys and water, this organ seems to be a promising endpoint for assessing the presence and potential effects of manganese in drinking water sources from the Bor area.

Regarding non-essential TMs, the kidney was the most sensitive organ to cadmium, showing a trend towards a dose-dependent increase with increasing levels in water. Indeed, literature data render this organ as a major site for Cd storage and toxicity [4,17,48,88]. However, the kidneys did not fulfill the abovementioned requirements needed to be considered a relevant biomarker of exposure to this TM. This may arise from the relatively low cadmium concentrations in water sources analyzed [88].

The brain and stomach were the most responsive organs to the increase in water lead. This TM has a half-life of about 30 days in the blood, after which it passes into soft tissues, including the kidneys and brain, before being distributed to bones, teeth, and hair as lead phosphate [17,18]. Given the marked linear relationships of the corresponding regression models, these organs seem to be promising endpoints for studying the impact of water contamination with lead on native terrestrial fauna.

### 4.5. Limitations, Strengths, and Future Research Directions

Several limitations of this exploratory study need to be acknowledged. First, we did not investigate the effects of water TMs on other relevant tissue-level endpoints, such as histopathological changes or enzymatic activities. However, the present results provide a comprehensive data set on the levels of various TMs in water sources from the Bor mining area and organs of male Wistar rats. These findings expand the little knowledge available on environmental TMs and native mammals; we investigated the distribution and retention of the same TMs in the heart, lungs, liver, spleen, kidneys, and testes of rats of similar age, variety, and sex exposed to metal-contaminated water collected at different sites [4]. Importantly, TM concentration measurements are applicable across a range of organisms, from small rodents to larger mammals, making them a versatile and widely applicable initial assessment method [17]. By measuring TM concentrations first, we also provide a reference point to understand the initial exposure levels and a solid foundation on which to conduct future, more complex environmental risk analyses. In addition, the present findings could help identify target organs that are particularly prone to TM accumulation [48,79].

Second, we did not determine pH and physico-chemical properties of water samples analyzed although these data are important for characterizing their quality. Certain TMs, such as Cu and Fe, were found here at very high levels in water samples at all sites, excepting the reference site. It is hence plausible that these TMs existed mainly in particulate, not dissolved, form [10,17]. These differences may significantly affect the responses of rats to water TM exposure [17,18]. As a result, future studies will include a thorough analysis of water samples, focusing on pH, dissolved oxygen levels, temperature, conductivity, and other physicochemical properties. Understanding these parameters can provide a clearer picture of the water quality and the form in which TMs exist.

Third, one has to take into account the exploratory character of the present study for understanding the practical relevance of water-to-organ regressions identified here. Particularly, this includes the selection of a single exposure period (thus, only one time point for analysis) and the chemical composition of drinking water from different sources. A linear regression analysis, as used here to identify the main target organs for different TMs, determines significant associations but does not establish a clear causation [26]. In fact, other factors, such as diet or metabolism, may play a role [17,79]. Since we used here specimens of similar age and size reared under the same experimental conditions, it is likely that the potential influence of the aforementioned factors was very limited.

Overall, the present findings should help environmental scientists in assessing the severity of contamination in drinking-water sources from the Bor area and mapping the spatial distribution of TM contamination. This is important for identifying vulnerable communities and prioritizing mitigation efforts. Several avenues for future research, including health assessments, mechanistic studies, environmental management, and regulatory considerations, should be addressed to better understand and address TM contamination in drinking water in this region and its potential implications for human and environmental health. For example, understanding how changes in water TM levels correspond to changes in organ TM concentrations may offer pertinent predictive capabilities for assessing TM retention in specific organs under varying exposure scenarios. An important topic of complementary research is the investigation of TM contamination (pollution) in soils exposed to these waters and the identification of potential remediation solutions. Use of organic–inorganic mixed improvers and various plants serves as a potential solution for remediation of TM-contaminated soils [89,90]—which is also the case of soils exposed to TM-contaminated waters from the Bor area [10,12].

## 5. Conclusions

Contamination (pollution) of surface waters with TMs resulting from mining and metal processing activities is a major threat for environmental health. Finding a solution to this problem is an important mission for scientists, which involves not only identifying the proper remedial actions and preventive measures, but also understanding the effect of this environmental insult on different organismal endpoints. Quantification of TM amounts in animal organs provides pertinent information about the initial exposure levels. Understanding TM impact on intra-organ mineral homeostasis and identification of the main target organs for each TM expand this knowledge, being essential for critical evaluation of TM-related risks in specific organs and under varying exposure scenarios. In our study, combined application of an ANOVA and regression analysis of TM levels in four water sources and eight rodent organs shows that

- TM-contaminated water sources from the Bor mining area can alter endogeneous TM levels across a large number of rat organs. Such changes may serve as more sensitive endpoints for such exposure events than body weight or survival.
- Perturbation of trace metal homeostasis is most evident in the brain, stomach, kidneys, and testes. The dynamics and magnitude of these imbalances depend on the target organ, trace metal analyzed, and water origin.
- Cadmium may be more potent than lead in modulating the changes in intra-organ concentrations of essential trace metals (copper, zinc, iron, and manganese).
- From an ecotoxicological point of view, the rat kidneys may serve as the most appropriate organ for manganese exposure assessment in the case of watercourses from the Bor mining area. Similar conclusions can be derived with respect to the use of the stomach and brain for monitoring changes in water lead.

Therefore, research should be expanded to encompass a larger array of animal groups, including invertebrates, fish, and birds. It is also crucial to determine the potential health risks to the local population originating from exposure to vegetables sprinkled with water from these sources, animals fed with them, and aquatic products in order to ensure their safety. The environmental/public health concerns related to water contamination/pollution in the Bor mining area can be better understood and managed by conducting such studies and extending the investigation to underground water sources.

## Figures and Tables

**Table 1:** TM levels in water sources analyzed.

Site	Zn (ppm)	Cu (ppm)	Fe (ppm)	Mn (ppm)	Cd (ppm)	Pb (ppm)
P1	0.05 (0.01)	nd	1.26 (0.44)	0.05 (0.03)	0.01 (0.01)	0.03 (0.02)
P2	0.08 (0.03)	117.76 (12.21)	305.76 (54.87)	7.98 (4.45)	0.03 (0.01)	1.55 (0.43)
P3	0.15 (0.06)	251.37 (32.77)	439.58 (43.17)	35.18 (7.13)	0.01 (0.01)	5.98 (1.02)
P4	0.21 (0.05)	475.68 (27.34)	511.77 (35.89)	68.78 (10.29)	0.11 (0.05)	16.77 (3.25)

Zn, zinc; Cu, copper; Fe, iron; Mn, manganese; Cd, cadmium; Pb, lead; nd, not detectable. Trace metal concentrations are expressed as parts per million dry weight (ppm) and are shown as averages with one standard deviation (in parentheses).

**Table 2:** Intra-organ TM Correlations.

	**Zn-Cu**	**Zn-Fe**	**Zn-Mn**	**Zn-Cd**	**Zn-Pb**	**Cu-Fe**	**Cu-Mn**	**Cu-Cd**
Brain	−0.81 ***	0.49	0.16	0.22	0.40	−0.80 ***	0.21	0.04
Heart	0.62 *	0.69 *	0.15	0.04	0.65 *	0.86 ***	0.15	−0.033
Lungs	0.76 **	−0.01	0.65 *	0.66 **	0.08	0.45	0.61 *	0.47
Stomach	0.95 ***	0.44	0.76 **	0.78 **	0.66 *	0.30	0.79 **	0.73 **
Liver	0.74 **	0.71 **	0.63 *	0.50	0.33	0.58 *	0.39	0.65 *
Spleen	−0.08	−0.90 ***	0.31	−0.14	0.14	−0.03	0.67 *	0.49
Kidneys	0.98 ***	0.95 ***	0.54	0..05	0.55	0.98 ***	0.55	0.09
Testis	0.90 ***	0.88 ***	0.83 **	0.96 ***	0.42	0.97 ***	0.96 ***	0.84 **
	**Cu−Pb**	**Fe−Mn**	**Fe−Cd**	**Fe−Pb**	**Mn−Cd**	**Mn−Pb**	**Cd−Pb**	
Brain	0.02	−0.63 *	−0.44	−0.45	0.87 ***	0.88 ***	0.79 **	
Heart	0.08	0.18	−0.45	0.06	0.22	0.15	0.55	
Lungs	0.49	−0.25	−0.08	0.36	0.23	0.31	−0.23	
Stomach	0.80 **	−0.07	0.49	−0.08	0.46	0.80 **	0.49	
Liver	0.54	0.29	0.46	0.72 **	0.19	−0.01	0.64 *	
Spleen	0.53	−0.20	0.24	−0.11	0.68 **	0.78 **	0.77 **	
Kidneys	0.68 *	0.45	0.01	−0.72 **	0.76 **	0.21	−0.04	
Testis	0.53	0.95 ***	0.86 ***	0.53	0.80 **	0.68 *	0.34	

Zn, zinc; Cu, copper; Fe, iron; Mn, manganese; Cd, cadmium; Pb, lead. Marked values (*) indicate significant correlations (Pearsons’s correlation, ***—*p* < 0.001, **—*p* < 0.01, *—*p* < 0.05).

**Table 3:** Water-to-organ Correlations and Regressions.

Site	Zn	Cu	Fe	Mn	Cd	Pb
Brain	0.88 (0.64) ***	−0.30	0.12	0.73 **	0.74 **	0.88 (0.77) ***
Heart	0.22	0.06	0.43	−0.03	0.07	−0.22
Lungs	−0.01	0.01	0.47	−0.37	−0.35	0.30
Stomach	0.72 (0.52) **	0.79 (0.62) ***	0.50	0.71 (0.50) **	0.34	0.88 (0.77) ***
Liver	−0.03	−0.55	0.34	0.23	−0.64 (0.41) **	−0.74 (0.55) **
Spleen	0.35	−0.35	−0.11	0.58 (0.34) *	0.47	0.46
Kidneys	−0.18	−0.63 (0.40) **	−0.80 (0.64) ***	0.87 (0.76) ***	0.76 (0.58) **	−0.28
Testis	0.17	0.06	−0.79 (0.62) **	−0.54	−0.17	−0.04

Zn, zinc; Cu, copper; Fe, iron; Mn, manganese; Cd, cadmium; Pb, lead. Data are shown as Pearsons’s correlations with the coefficients of regressions of the corresponding water-to-organ regressions (in parenthesis). Marked values (*) indicate significant correlations (Pearsons’s correlation, ***—*p* < 0.001, **—*p* < 0.01, *—*p* < 0.05).

## Data Availability

The original data involved in this study have been presented in the paper.
